# Comparative performance assessment of deep learning based image steganography techniques

**DOI:** 10.1038/s41598-022-17362-1

**Published:** 2022-10-07

**Authors:** Varsha Himthani, Vijaypal Singh Dhaka, Manjit Kaur, Geeta Rani, Meet Oza, Heung-No Lee

**Affiliations:** 1 Department of Computer and Communication Engineering, Manipal University Jaipur, Jaipur, 303007 India; 2 School of Electrical Engineering and Computer Science, Gwangju Institute of Science and Technology, Gwangju, 61005 Korea

**Keywords:** Electrical and electronic engineering, Computer science, Information technology

## Abstract

Increasing data infringement while transmission and storage have become an apprehension for the data owners. Even the digital images transmitted over the network or stored at servers are prone to unauthorized access. However, several image steganography techniques were proposed in the literature for hiding a secret image by embedding it into cover media. But the low embedding capacity and poor reconstruction quality of images are significant limitations of these techniques. To overcome these limitations, deep learning-based image steganography techniques are proposed in the literature. Convolutional neural network (CNN) based U-Net encoder has gained significant research attention in the literature. However, its performance efficacy as compared to other CNN based encoders like V-Net and U-Net++ is not implemented for image steganography. In this paper, V-Net and U-Net++ encoders are implemented for image steganography. A comparative performance assessment of U-Net, V-Net, and U-Net++ architectures are carried out. These architectures are employed to hide the secret image into the cover image. Further, a unique, robust, and standard decoder for all architectures is designed to extract the secret image from the cover image. Based on the experimental results, it is identified that U-Net architecture outperforms the other two architectures as it reports high embedding capacity and provides better quality stego and reconstructed secret images.

## Introduction

Image encryption and image steganography are the most common ways to secure image data. In image encryption, the image is encoded using an encryption technique^1^. In image steganography, an image is embedded in some cover media such as image, audio, video, etc*.*^2^. The advantage of image steganography is that it is hard to distinguish that a secret image is hidden into the cover media^3^. Whereas in image encryption, encrypted images are noise-like that may attract an attacker.

The traditional steganography technique ‘Least Significant Bit’ (LSB) substitutes the secret data bits on LSBs of image pixel values^2,3^. But it leaves traces of hidden data that can be detected by steganalysis^4,5^. To enhance security, many improvements to the LSB technique were proposed^5–8^. However, there is no significant improvement observed in the essential properties of steganography in these techniques^5–8^. To provide better results than LSB, transform domain-based steganography techniques were proposed^9–13^. In these techniques, the secret data is embedded into coefficient values. However, these techniques suffer from low payload capacity and poor visual quality of stego and reconstructed images^9,10^.

To improve the weak aspects of the above-discussed methods, machine learning-based steganography methods such as the Genetic algorithm^14–16^ and fuzzy logic-based^17,18^ were proposed. These techniques have significantly improved the visual quality of the stego and reconstructed image, but the flaws like high complexity and low payload capacity are not elucidated. To improve security, support vector machine-based steganography techniques^19,20^ is proposed, but these techniques are not suitable for large datasets.

In recent years, image steganography based on convolutional neural network (CNN) has gained wide research attention due to its superior capabilities against traditional methods^21^. In these methods, the secret image is embedded into cover media by intelligent and accurate coefficient selection. It enhances the performance of steganography in all the aspects like payload capacity, imperceptibility, and reconstructed image visual quality, etc*.*^22^.

A CNN-based image steganography technique proposed by Rehman et al*.* improved the visual quality of the stego image by hiding the gray-scale secret image in specific extracted features of the color cover image^23^. Further, Baluja proposed an autoencoder and decoder scheme^24^. In this, three networks are prepared, first is the preparation network that transforms the RGB pixels of the secret image into features. The second is a hiding network that hides the features obtained by the preparation network into the cover image. The third is the reveal network, which extracts the secret image from the cover image. Here, the payload capacity and stego image visual quality are improved, but the visual quality of the reconstructed image is significantly compromised. Duan et al*.* increased the payload capacity by embedding two secret images in one cover image^25^.

Further, Zhang et al*.* proposed the improvement in the stego image visual quality by converting the cover image in YCrCb format^26^. Only the ‘Y’ channel is used to hide the secret grayscale image without affecting the ‘Cr’ and ‘Cb’ channels. These two channels contain all the color information. Hence, the stego image quality is improved, but this method is limited to a secret grayscale image. The U-net architecture-based steganography is proposed to improve the payload capacity and reconstructed image quality^27,28^. Wu et al*.* proposed CNN-based steganography that enhanced the payload capacity and stego image quality^29^. For further improvements, steganography techniques based on the generative adversarial networks are proposed^30–33^. These networks generate high-quality stego and reconstructed images at a low computation cost. However, the security of these methods also needs improvement. Table 1 provides the summary of the existing literature discussed above.

**Table 1:** Summary of the existing literature.

References	Steganography method	Improved	Needs to be improved
^2,3,5–8^	Traditional LSB based	Easy implementation	Security, payload capacity, visual quality of stego image and recovered image
^9–13^	Transform domain based	Better security and payload capacity than traditional LSB	Visual quality of stego and reconstructed images
^14–18^	Machine learning based	Better visual quality of stego and reconstructed images	High complexity, payload capacity can be improved
^19,20^	Support vector machine based	Better security	Not suitable for large dataset
^23–29^	CNN based	High payload capacity, reconstruction quality	Computational cost, security from deep learning based steganalysis
^30–33^	GAN based	High visual quality stego and reconstructed images, low computation cost	Security from deep learning based steganalysis

In recent literature, Sharma et al*.* proposed an image steganography technique based on graph signal processing. In this method, the secret image is first scrambled through quantum scrambling to enhance the security. Then, both the cover image and secret image are transformed by graph wavelet transform that improved the visual quality of the stego image and recovered the secret image^34^. Shen et al*.* presented an image steganography technique for the applications based on wireless visual sensor networks by using partial preservation embedding algorithm^35^. Telli et al*.* proposed a multi-image steganography technique inspired by Baluja’s scheme^24^ and improved stego image visibility^36^. Peter et al*.* improved the payload capacity of the steganography by using the histogram shifting method and quick response decomposition method^37^.

It is evident from the literature that CNN-based steganography has the potential of securing image data by hiding the secret image into a cover image. But, each CNN-based architecture requires its unique corresponding decoder to decode the secret image at the receiver end. Furthermore, there is enormous scope to enhance the quality of reconstructed images, improve the payload capacity and reduce the computation time.

In this work, performance analysis of CNN based on three deep learning architectures *i.e.*, U-Net, V-Net, and U-Net++ for steganography is carried out. U-Net architecture for image steganography is analyzed in the literature^27^. However, its efficacy is not envisaged with similar CNN based techniques like V-Net and U-Net++ architectures. In this paper, V-Net and U-Net++ architectures are first time implemented for image steganography and their performance compared to U-Net is analyzed.

The main contribution of this paper could be summarized as follows:

- A comparative assessment is carried out between U-Net, V-Net, and U-Net++ architecture-based steganography, and various performance parameters are evaluated.
- Three image-in-image steganographic techniques based on U-Net, V-Net, and U-Net++ are proposed for confidential communication and storage of data.
- Developed a deep learning-based decoder that can decode the stego images generated by either of the three proposed encoders.

The proposed architectures hide a secret image of dimension N × N into a cover image of the same dimensions. In contrast to the methods^23,24,26^, this research employs U-Net, V-Net, and U-Net++ architectures as encoders to hide the secret image into the cover image and a common decoder architecture to extract secret image from the stego image generated by either of the used encoder architectures.

Best of the authors’ knowledge, implementation of V-Net and U-Net++ architectures for image steganography and comparative performance assessment of U-Net, V-Net, and U-Net++ encoders are not reported in the literature and can be considered as the unique contribution of the proposed work.

In the remaining part of the paper, “Background” section describes the background of the CNN architectures; “Proposed methodology” section demonstrates the proposed methodology. “Evaluation metrics” section gives the details of the experimental details. “Results and discussion” section discusses the implications of the proposed work and its performance supremacy over the related works. Finally, “Conclusions” section presents the conclusions of the proposed work.

## Background

CNN architectures gained popularity due to automatic feature extraction, reduced feature map, high accuracy, and versatile application areas^38^. The potential of CNN architectures is proved in the applications of pattern recognition^39^, classification^40^, object recognition^41^, and image segmentation^42,43^. In recent literature, the applications of these networks are also observed in steganography^9^. U-Net, V-Net, and U-Net++ are widely known architectures used for image segmentation. In this paper, the applications of these architectures are extended in image steganography.

### U-Net

**Figure 1:**
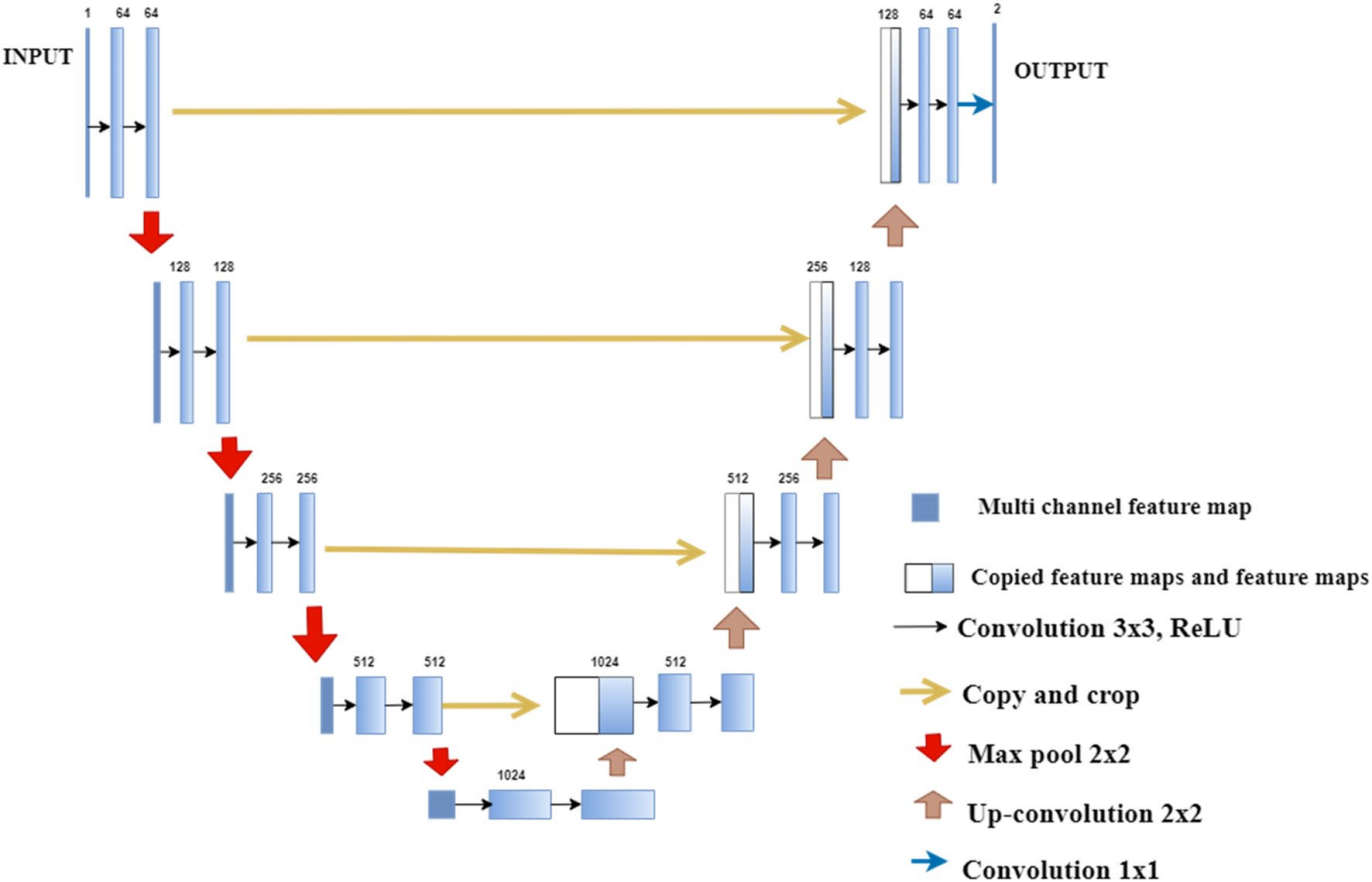
U-net architecture.

U-Net is a fully convoluted neural network that provides enhanced performance with fewer training images^42^. Figure 1 shows the example of U-Net architecture^42^. The blue-colored boxes represent the multi-channel feature maps. This architecture consists of contraction (convolution) and expansion (deconvolution) paths. Each path comprises 23 convolutional layers. Each layer of the contraction path contains two filters of dimensions 3 × 3 that repeatedly perform unpadded convolutions. The feature channels are doubled in each convolution layer.

Further, each convolution operation is followed by the 2 × 2 max-pooling operation. The expansion path of the encoder performs deconvolution operations by using the filter size of 2 × 2. The feature channels are halved in each deconvolution layer. Thus, horizontal connections from the left to the right path to forward extracted features at the early stages. This improves the quality of the final reconstructed image by providing spatial information lost during contractions^42^.

### V-Net

**Figure 2:**
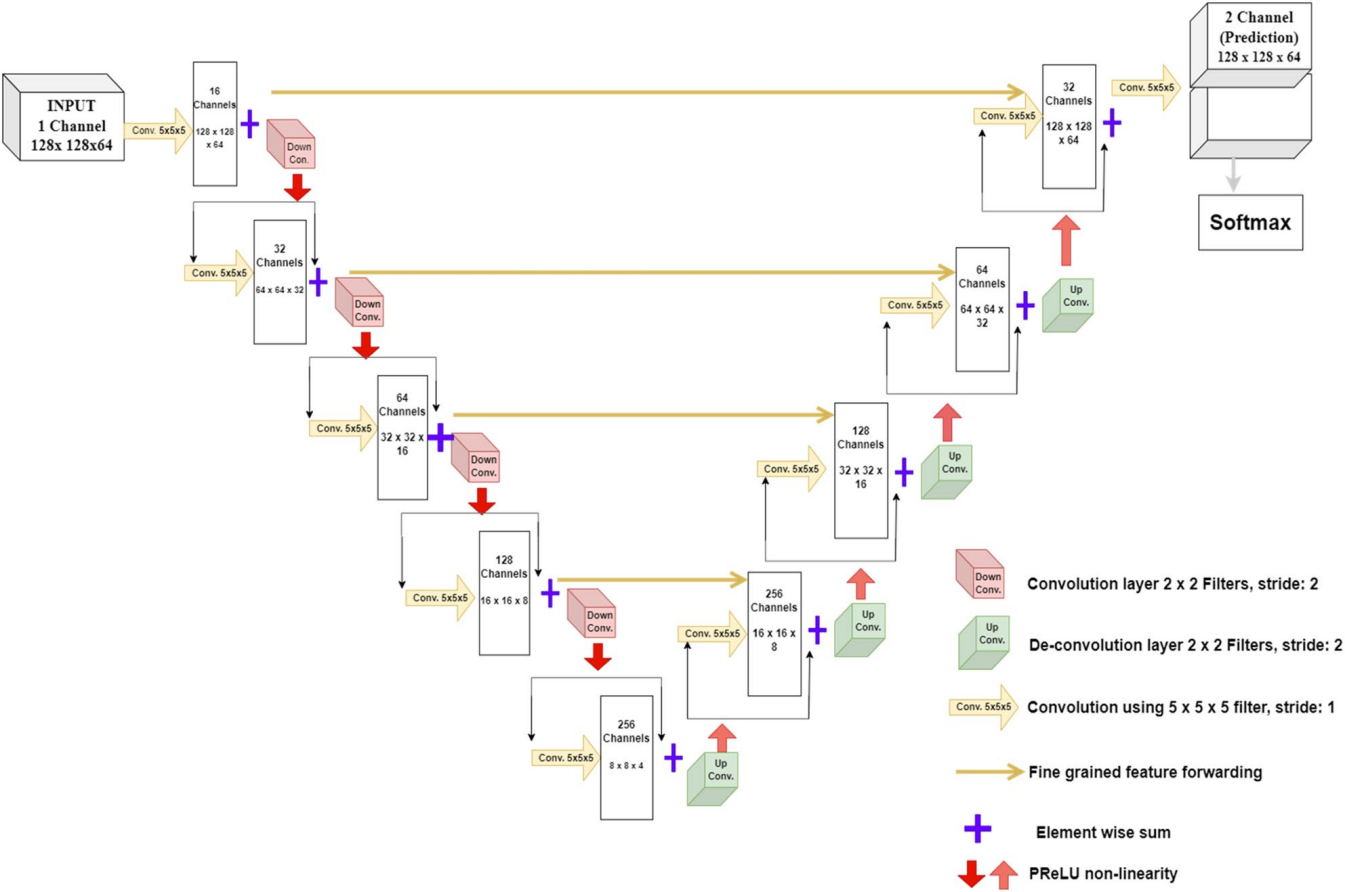
V-net architecture.

The V-Net convolutional network is specially designed to take volumetric inputs. This architecture is **s**imilar to U-Net based architecture except that the contraction path has 1–3 convolution layers in each stage and substitutes max pooling operations with the convolution operations. Figure 2 represents the example of V-Net architecture. The network is divided into phases to work with volumetric inputs in the contraction path, and extracted features are expanded in the expansion path. In contrast to U-Net, the residual function is learned at each stage of contraction and expansion path that ensures convergence of the architecture^43^.

### U-Net++

The U-Net++ architecture is the tailored version of the U-Net. In this architecture, convolution layers are on skip pathways that tie the semantic gap among encoder and decoder feature maps. The count of convolution layers is dependent on the skip pathways. The number of skip pathways is calculated by using Eq. (1)^44^. These skip pathways connect the two sub-networks for deep supervision. A concatenation layer follows each convolution layer in the dense convolution block. The concatenation layer provides the output obtained by the fusion of the current and its previous convolution layers' outputs.

1 $${S}^{i,j}= \left\{\begin{array}{ll}  Con\left({S}^{i-1, j}\right) ,&\quad j=0\\ Con([{\left[{S}^{i, k}\right]}_{k=0}^{j-1} , De{(S}^{i+1, j-1})]) ,&\quad j>0\end{array}\right.$$

In Eq. (1), the function $con$ is the convolution operation, function $De$ is the deconvolution operation. Here, ${S}^{i,j}$ denotes the stack of feature map of the node ${s}^{i,j}$, $i$ is the index of the contraction layer, $j$, and $k$ are the indices of the convolution layers of the dense block. The dense skip connections on skip pathways enhances the gradient flow.

**Figure 3:**
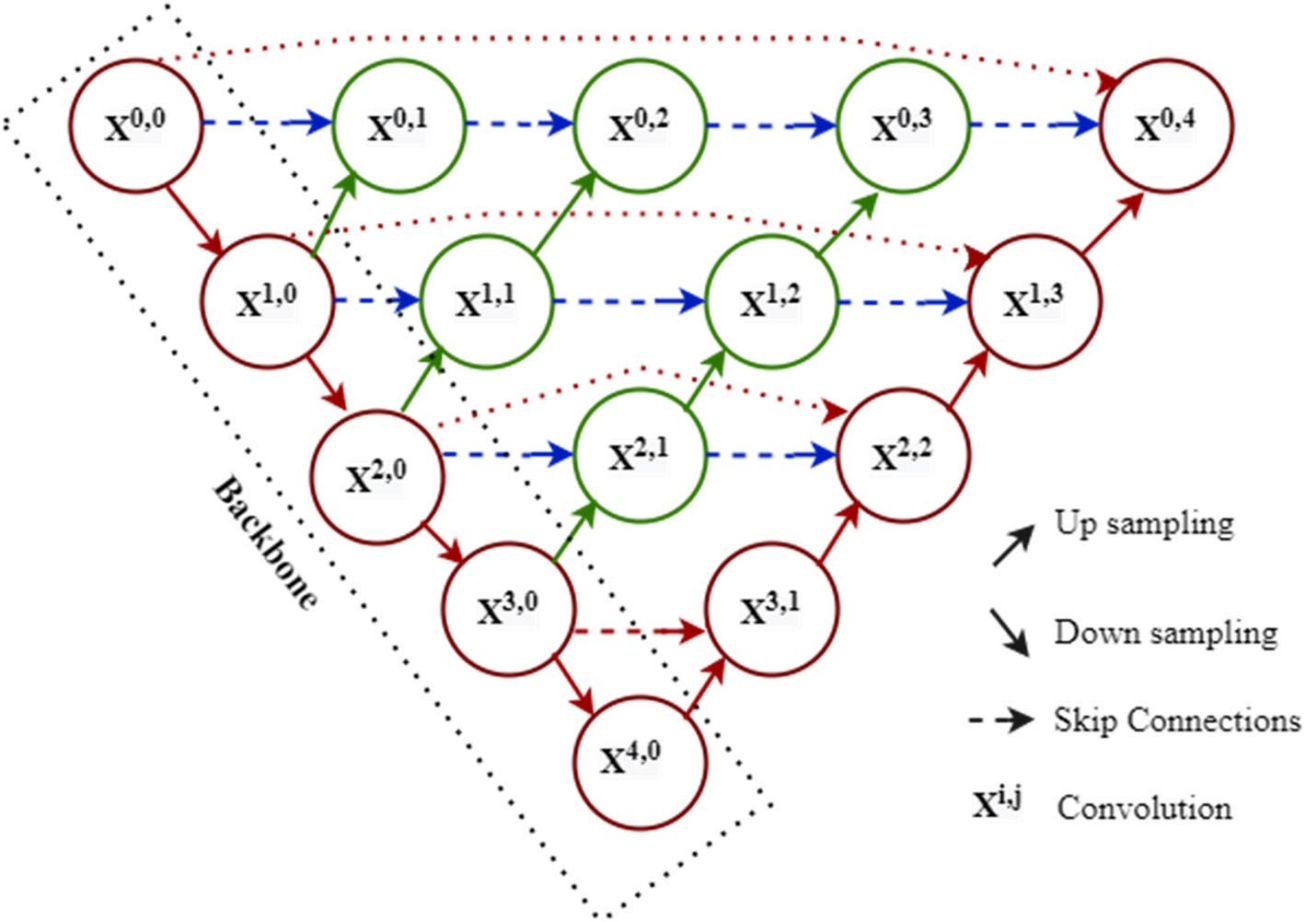
U-Net++ architecture.

Also, the U-Net++ allows flexible network depth and is free from unnecessary limiting skip connections. Here, the merging of same-scale feature maps is considered. Further, the U-Net++ architecture allows compact feature proliferation through the compactly associated skip connections. Therefore, at the decoder nodes, more flexible feature fusion is obtained. The multiscale feature aggregation leads to deep supervision, high accuracy, and fast convergence. Figure 3 illustrates the example of U-Net++ architecture^44^. The red lines show the original U-Net architecture. In U-Net++ convolution layers are on skip pathways, that draw the semantic gap between encoder and decoder feature maps. The blue and green lines represent the dense skip connections on skip pathways.

## Proposed methodology

**Figure 4:**
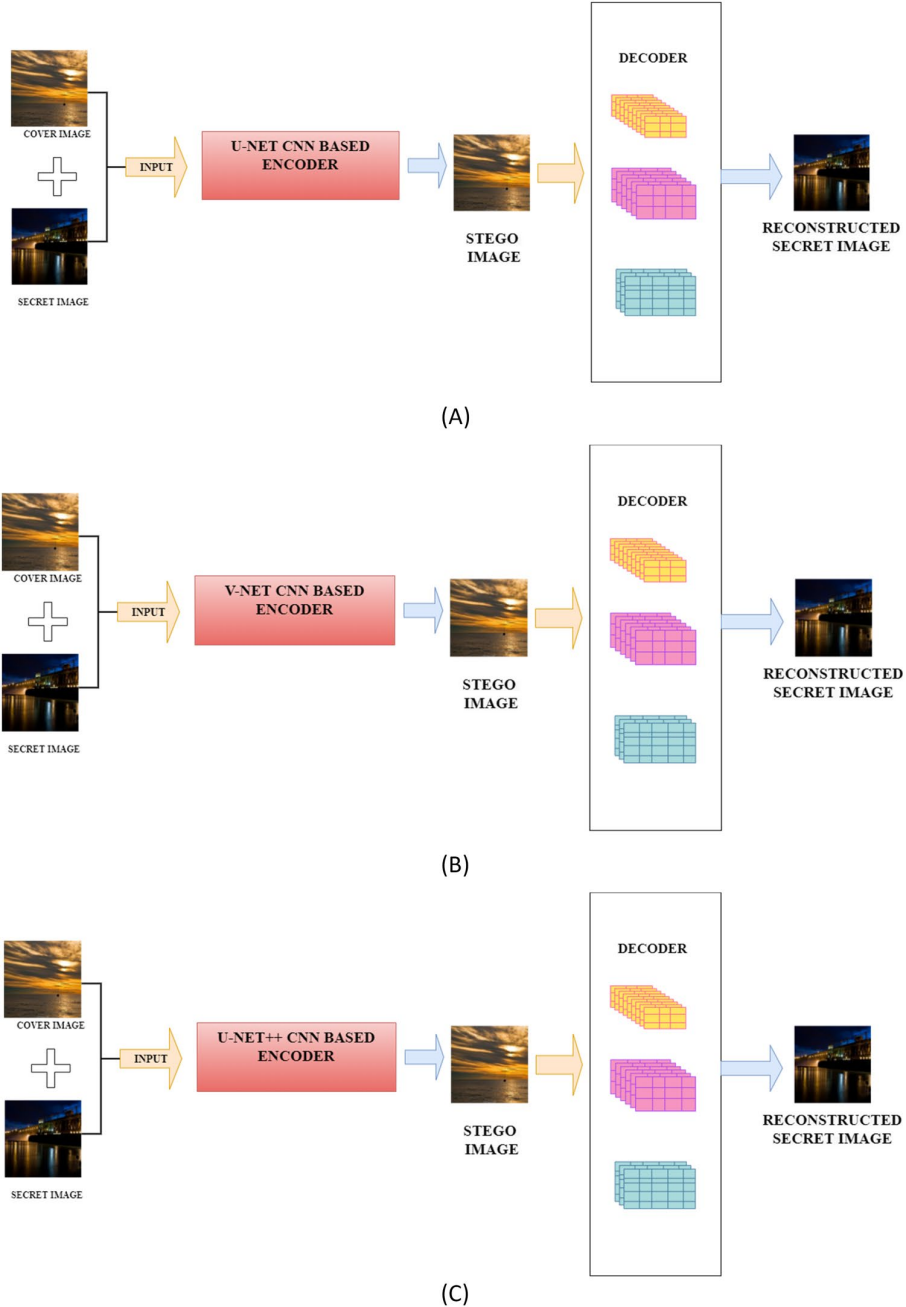
Block diagram of the proposed steganography techniques (**A**) U-Net architecture based encoder; (**B**) V-Net architecture based encoder; (**C**) U-Net++ architecture based encoder.

In this work, deep learning-based image-in-image steganography techniques are implemented and their performance is assessed. The architecture shown in Fig. 4 demonstrates three deep learning architectures, viz*.* U-Net, V-Net, and U-Net++ based encoders that are employed to hide secret image into the cover image.

A unique decoder architecture is designed to extract hidden secret image from the stego image. The architectural details of the encoders and decoder are illustrated in the subsequent subsections 3.1 and 3.2, respectively.

### Architecture of encoders

In the proposed steganography techniques, three fully connected distinct CNN architectures viz*.* U-Net^42^, V-Net^43^, and U-Net++^44^ are implemented to generate a stego image that hides the secret image into the cover image.

### Architecture of decoder

As a part of this research, a CNN-based unique and robust decoder is designed. The purpose of the decoder is to extract secret images from the stego images generated by any of the U-Net, V-Net, and U-Net++ based encoders.

**Figure 5:**
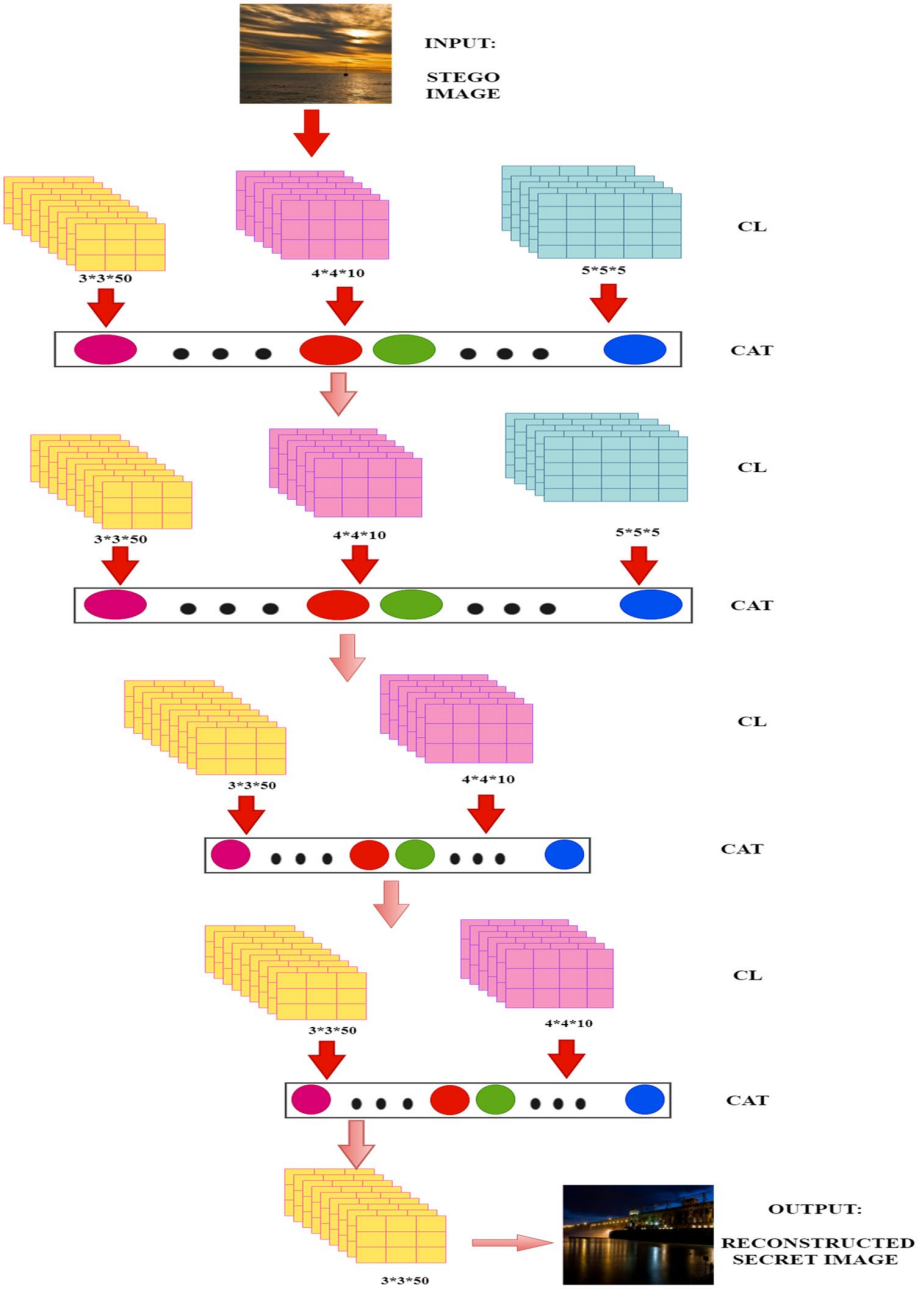
The architecture of the decoder network.

As shown in Fig. 5, the decoder contains 11 convolution layers with different kernel sizes of 3 × 3, 4 × 4, and 5 × 5. Each kernel has multiple filters for enhancing the feature extraction capabilities of the decoder network. The convolution (CL) layers provide the feature maps as their outputs. The concatenation (CAT) of convolutional layers is performed to extract essential and useful semantic features from the feature maps^45^. These features improve the learning of the model. The details of the output size and number of parameters at each layer are demonstrated in Table 2. In this, the first column gives the details of each layer of the network where *conv* is the convolution, and *level* shows the level of the convolution layer. The second column shows the output size obtained corresponding to each input layer. The last column indicates the number of parameters at each layer of the network.

**Table 2:** Structure of the decoder network .

Layer (type)	Output size	Parameters
Input Layer	256, 256 ,3	0
conv_level0_3 × 3 (Conv2D)	256, 256, 50	1400
conv_level0_4 × 4 (Conv2D)	256, 256, 10	490
conv_level0_5 × 5 (Conv2D)	256, 256, 5	380
Concatenate_conv_level0	256, 256, 65	0
conv_level1_3 × 3 (Conv2D)	256, 256, 50	29,300
conv_level1_4 × 4 (Conv2D)	256, 256, 10	10,410
conv_level1_5 × 5 (Conv2D)	256, 256, 5	8130
Concatenate_conv_level1	256, 256, 65	0
conv_level2_3 × 3 (Conv2D)	256, 256, 50	29,300
conv_level2_4 × 4 (Conv2D)	256, 256, 10	10,410
Concatenate_conv_level2	256, 256, 60	0
conv_level3_3 × 3 (Conv2D)	256, 256, 50	27,050
conv_level3_4 × 4 (Conv2D)	256, 256, 10	9610
Concatenate_conv_level3	256, 256, 60	0
conv_level4_3 × 3 (Conv2D)	256, 256, 50	27,050
Output Layer	256, 256, 3	1353

### Training

In this research, the training and testing of the proposed architecture are carried out on a machine with RTX 2,080 Graphics Processing Unit (GPU) with 96 GB RAM and a 2 TB hard disk. The GPU runs with Ubuntu 16.04 operating system.

#### Dataset preparation

The dataset used for training the encoders and decoder is available online^46,47^. The dataset comprises 6616 color images with 3 channels and dimensions of 256 × 256. To ensure robustness, the model is trained on the imagery dataset of different types.

The test dataset^46,47^ comprising 250 images of various kinds is used to evaluate the model performance. While training, each of the U-Net, V-Net, and U-Net++ based encoders individually take a cover and a secret image as inputs and provides a corresponding stego image as an output. The decoder network is trained simultaneously to extract the secret image from the stego image. Equation (2) shows the convolution operation performed at each convolution layer of the decoder.

2 $${Y}_{L}={\sum }_{x=0}^{N-1}{S}_{x}{K}_{L-x}$$

where, $${Y}_{L}$$ denotes the output of each convolution operation, $$S$$ is stego image data bits, $$K$$ is the kernel size, and $$N$$ is the number of elements in $$S$$. Multiple convolution operations are performed at each convolution layer to give a feature map ($${f}_{L})$$ as output. Now, $${f}_{L}$$ of all the *n* convolution layers $$L (L=1,\dots n)$$ are concatenated as given in Eq. (3).

3 $$CAT\left(i\right)=\frac{1}{{w}_{o}\times {h}_{o}} \sum {f}_{L} \left(.,.,i\right),\mathrm{ i}= 1, 2,\dots {\mathrm{c}}_{\mathrm{o}}$$

Here, ${c}_{o}$ denotes the number of channels in layer $L$, ${w}_{o}$ and ${h}_{o}$ are the width and height of the channel, respectively. Each convolutional layer is employed with Rectified Linear Unit (ReLU) activation function^48^. This function returns ‘0’ for the negative input and the same value ‘v’ for any positive input value ‘v’^44^. It is defined in Eq. (4).

4 $$ {\text{ReLU (v)}} = {\text{Maximum(0,v)}} $$

Further, the Adam optimizer is employed for providing computationally fast and efficient learning. This optimizer reduces the memory requirements in comparison to the classical stochastic gradient descent approach^49^. To carry out the training, the batch size of 32, 16, and 8 images are selected for the U-Net, V-Net, and U-Net++ based encoders, respectively. The batch size is selected based on the CNN models to maximize GPU utilization with minimum overhead.

The training parameters such as the exponential decay of the first and second moments of the gradients are set to 0.9 and 0.999, respectively. The value of another training parameter, ‘epsilon,’ is set to 1e-07^49^. In the contrast, the learning rate (alpha) is set to 0.0001, which is smaller than the value (0.001) used in the reference^49^. The value of the learning rate is decided based on the experiments conducted in this research. By varying the learning rate from 1 to 0.0001, it is observed that the model reported the minimum value of loss function and highest accuracy at the 0.0001 value of the learning rate. Further, it is witnessed that there is a slight decrease in the accuracy when the learning rate is increased from 0.0001 to 0.0005, but a sharp decline is observed on further increasing its value. The loss functions defined in Eqs. (5) and (6) are employed to train the encoder and decoder networks. This loss function is effective in reducing the training error in the process of embedding the secret image into the cover image.

In Eq. (5), $c,$ ${c}^{{\prime}}$ are the cover, and stego images, respectively. In Eq. (6), $s,$ and ${s}^{{\prime}}$ are the secret, and reconstructed secret images, respectively. Here, E is the image reconstruction error as defined in reference^31^.

5 $$lossencoder=\left|\left|c-{c}^{{\prime}}\right|\right|$$

6 $$lossdecoder=E\left|\left|s-{s}^{{\prime}}\right|\right|$$

At each epoch of training, the value of the loss function is computed. The weights of the network dynamically change until the value of the loss function approaches a minimum value. This ensures that the network is trained to generate a high-quality stego image. The stego image contains a secret image hidden into it. Still, it appears indistinguishable from the cover image. Simultaneously, the decoder is trained to extract the secret image from the stego image generated by any of the encoders. The loss function for the decoder is computed as the difference in the secret image and its corresponding reconstructed secret image.

The training procedure of the encoder and decoder network is illustrated in Algorithm 1. In step 1, the encoder receives the cover image $c$ and the secret image $s$ as inputs and hides the secret image $s$ into cover image $c$ to generate the stego image ${c}^{{\prime}}$. Now, at the second step, the value of the loss function is calculated as the difference between the original cover image $c$ and the stego image ${c}^{{\prime}}$, as defined in Eq. (5). In step 3, this loss function is backpropagated to the encoder network and the weights of the encoder are updated. Then, the encoder iterates steps 2 to 3 until the value of the loss function becomes negligible and the encoder is trained enough to generate the imperceptible stego image. Now, the decoder receives the stego image ${c}^{{\prime}}$ and extracts the secret image ${s}^{{\prime}}$ at the next step. Next, in step 6, the value of the loss function is computed as per Eq. (6). Now, in step 7, this value of the loss function is backpropagated to the decoder network and the weights of the decoder are updated. The procedure followed in steps 6 to 7 is repeated until the value of the loss function calculated for the decoder network becomes negligible. The decoder is trained enough to extract the secret image without degrading its quality. Now, the reconstructed secret image ${s}^{{\prime}}$ is obtained as the output image.
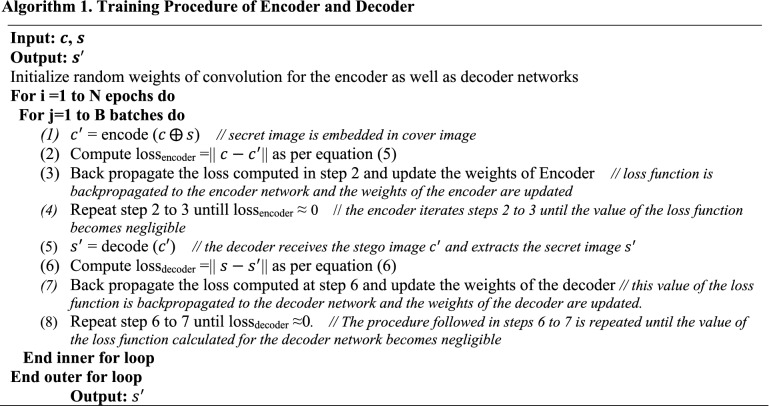


## Evaluation metrics

For evaluating the performance of the steganography techniques based on U-Net, V-Net, and U-Net++, the following metrics are used. These metrics are the measure of image quality as discussed in^50–52^. Thus, these are important for comparison in the quality of the images reconstructed by the steganography techniques.

### Mean square error (MSE)

This is the difference between the pixel values of secret and reconstructed images^53^ as defined in Eq. (7). In Eq. (7), $a$ and $b$ are image pixel coordinates with the size of $M\times N$ pixels. Here, ${I}_{1}$ and ${I}_{2}$ are original and reconstructed images, respectively^50^. Thus, the minimum value of MSE favors the better quality of the reconstructed image.

7 $$\mathrm{MSE}=\frac{{\sum }_{\mathrm{a}=1}^{\mathrm{a}=\mathrm{M}}{\sum }_{\mathrm{b}=1}^{\mathrm{b}=\mathrm{N}}[{\mathrm{I}}_{1}(\mathrm{a},\mathrm{b})-{\mathrm{I}}_{2}(\mathrm{a},\mathrm{b}){]}^{2}}{\mathrm{M}\times \mathrm{N}}$$

### Peak signal to noise ratio (PSNR)

It is the peak signal-to-noise ratio between the secret and reconstructed images. In Eq. (8), ${R}_{I}$ is the maximum variation in the input image data type, and $MSE$ is the mean square error. The value of PSNR is used to measure the visual quality difference between two images^51^. Its high value indicates the better quality of the reconstructed image.

8 $$\mathrm{PSNR}=10.{\mathrm{log}}_{10}\left(\frac{{\mathrm{R}}_{\mathrm{I}}^{2}}{\mathrm{MSE}}\right)$$

### Structural similarity Index (SSIM)

This metric is used to measure the deterioration in the image quality caused due to processing. It also measures the difference in the perceptual quality of the secret and reconstructed images. SSIM is the combined evaluation for the luminance ($l$), contrast ($c$), and the structure ($s$) of two images (a and b)^51^.

9 $$\mathrm{SSIM}(\mathrm{a},\mathrm{b})=[\mathrm{l}(\mathrm{a},\mathrm{b}){]}^{\mathrm{\alpha }}.[\mathrm{c}(\mathrm{a},\mathrm{b}){]}^{\upbeta }.[\mathrm{s}(\mathrm{a},\mathrm{b}){]}^{\upgamma }$$

In Eq. (9),

10 $$l\left(\mathrm{a},\mathrm{b}\right)=\frac{2{\upmu }_{\mathrm{a}}{\upmu }_{\mathrm{b}}+{\mathrm{C}}_{1}}{{\upmu }_{\mathrm{a}}^{2}+{\upmu }_{\mathrm{b}}^{2}+{\mathrm{C}}_{1}}, \mathrm{c}\left(\mathrm{a},\mathrm{b}\right)=\frac{2{\upsigma }_{\mathrm{a}}{\upsigma }_{\mathrm{b}}+{\mathrm{C}}_{2}}{{\upsigma }_{\mathrm{a}}^{2}+{\upsigma }_{\mathrm{b}}^{2}+{\mathrm{C}}_{2}},\mathrm{ s}\left(\mathrm{a},\mathrm{b}\right)=\frac{{\upsigma }_{\mathrm{ab}}+{\mathrm{C}}_{3}}{{\upsigma }_{\mathrm{a}}{\upsigma }_{\mathrm{b}}+{\mathrm{C}}_{3}}$$

where, ${\mu }_{a}$ and ${\mu }_{b}$ are the average of original and reconstructed images,${\sigma }_{a}$ and ${\sigma }_{b}$ are standard deviations, and ${\sigma }_{a.b}$ is covariance for images a and *b*. If $\alpha =\beta =\gamma =1$ and, ${C}_{3}=\frac{{C}_{2}}{2}$, SSIM can be simplified as:

11 $$S\mathrm{SIM}=\frac{\left(2{\upmu }_{\mathrm{a}}{\upmu }_{\mathrm{b}}+{\mathrm{C}}_{1}\right)\left(2{\upsigma }_{\mathrm{ab}}+{\mathrm{C}}_{2}\right)}{\left({\upmu }_{\mathrm{a}}^{2}+{\upmu }_{\mathrm{b}}^{2}+{\mathrm{C}}_{1}\right)\left({\upsigma }_{\mathrm{a}}^{2}+{\upsigma }_{\mathrm{b}}^{2}+{\mathrm{C}}_{2}\right)}$$

### Entropy

Entropy ($H\left(K\right)$) is the degree of uncertainty present in an image as defined in Eq. (12). In this equation, ${p}_{i}$ is the occurrence probability of the pixel $i$ in the image K. Entropy is used to quantify the information available in the image. More amount of information indicates better quality of image^52^.

12 $$H(K)=-{\sum }_{i=1}^{n}{p}_{i}{\mathit{log}}_{2}{p}_{i}$$

### Blind/reference less image spatial quality evaluator (BRISQUE SCORE)

This is used to estimate the perceptual quality of an image using the locally normalized luminance coefficients. In this manuscript, the BRISQUE SCORE as defined in the reference^48^ is used. It provides the no-reference image quality score by comparing the image to default natural scene images with similar distortions. The mean score is assigned between 0 and 100. A low score signifies better perceptual quality^53^.

## Results and discussion

### Image quality measures

**Figure 6:**
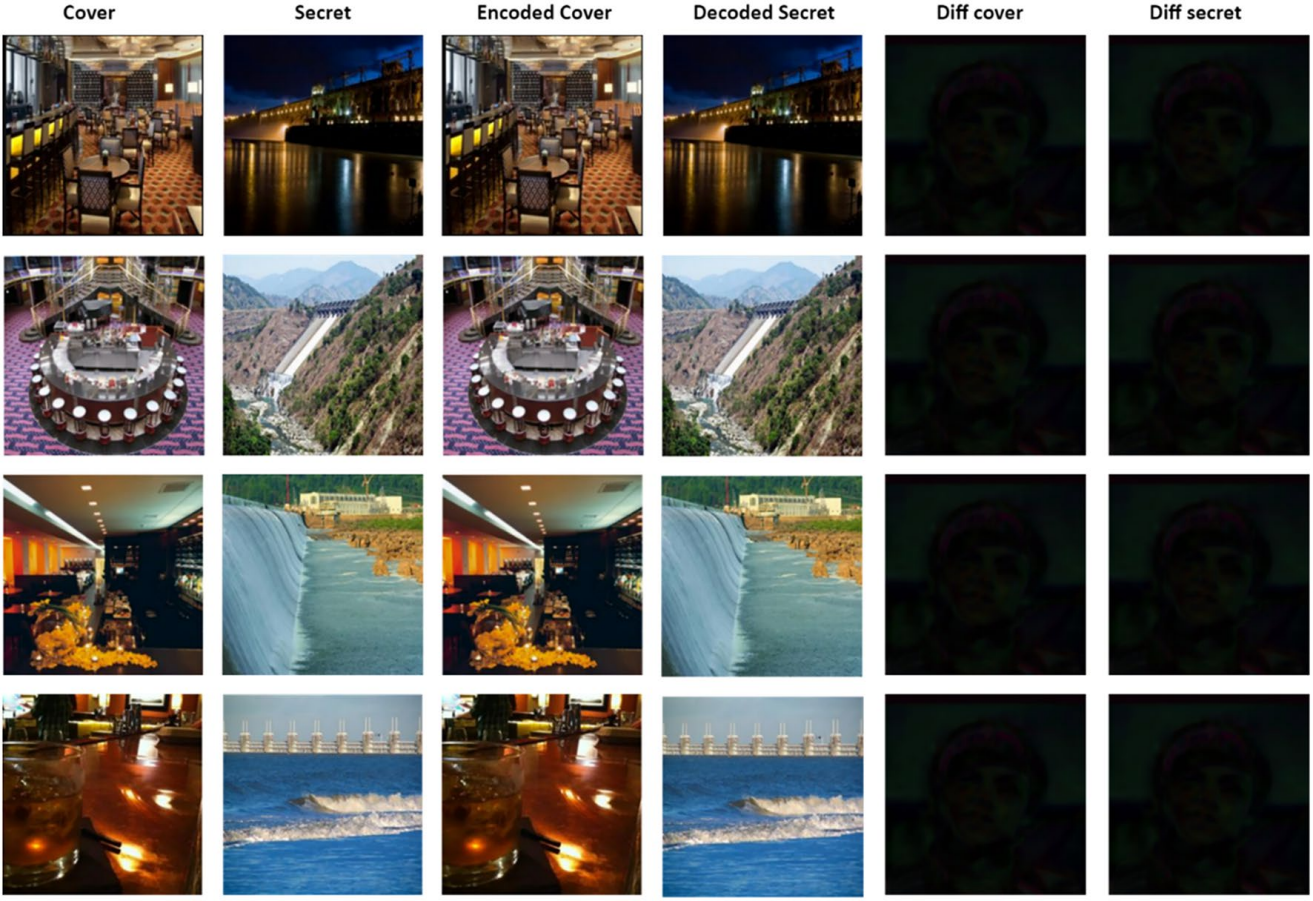
Test samples of U-Net encoder model.

**Figure 7:**
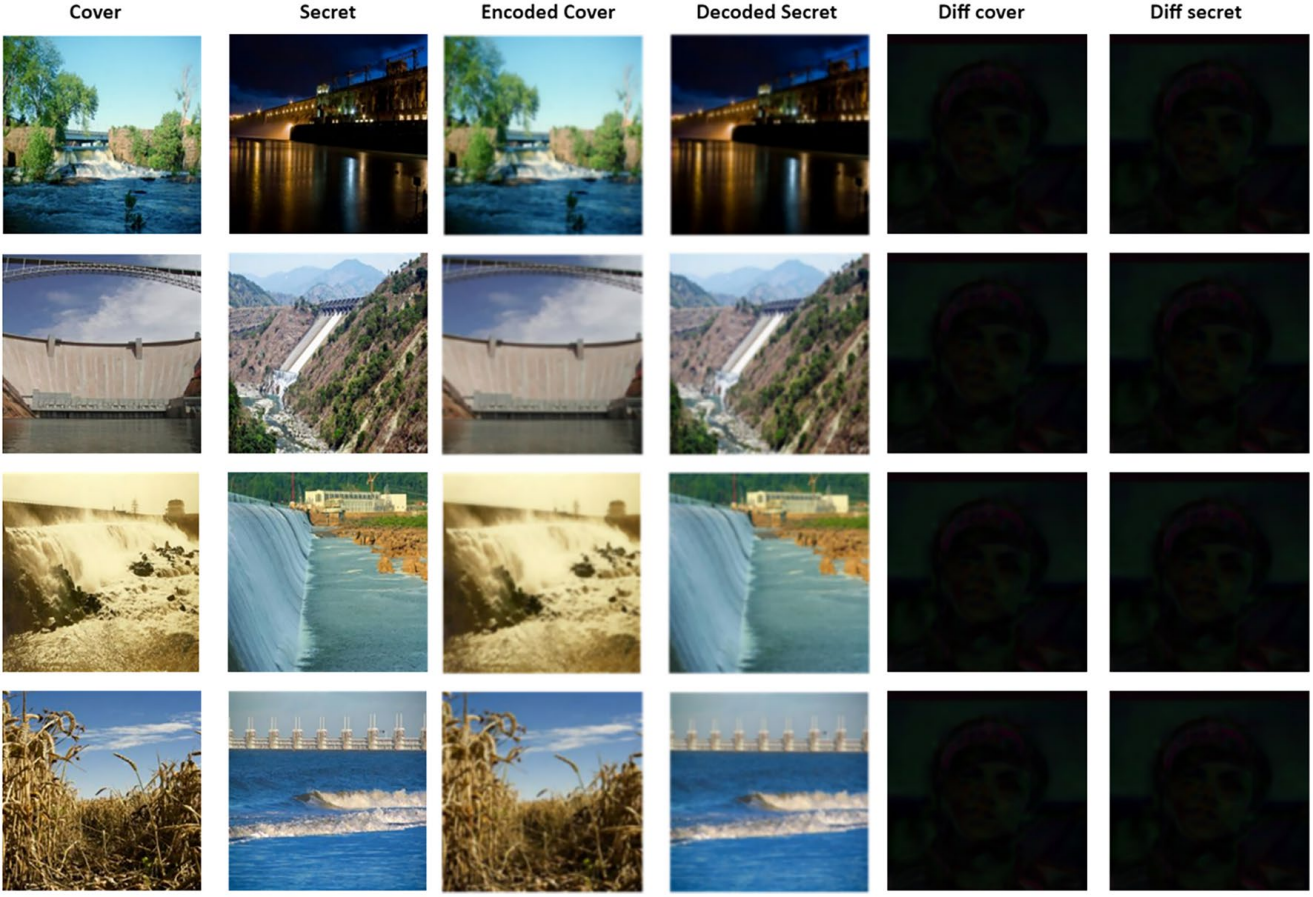
Test samples of V-Net encoder model.

**Figure 8:**
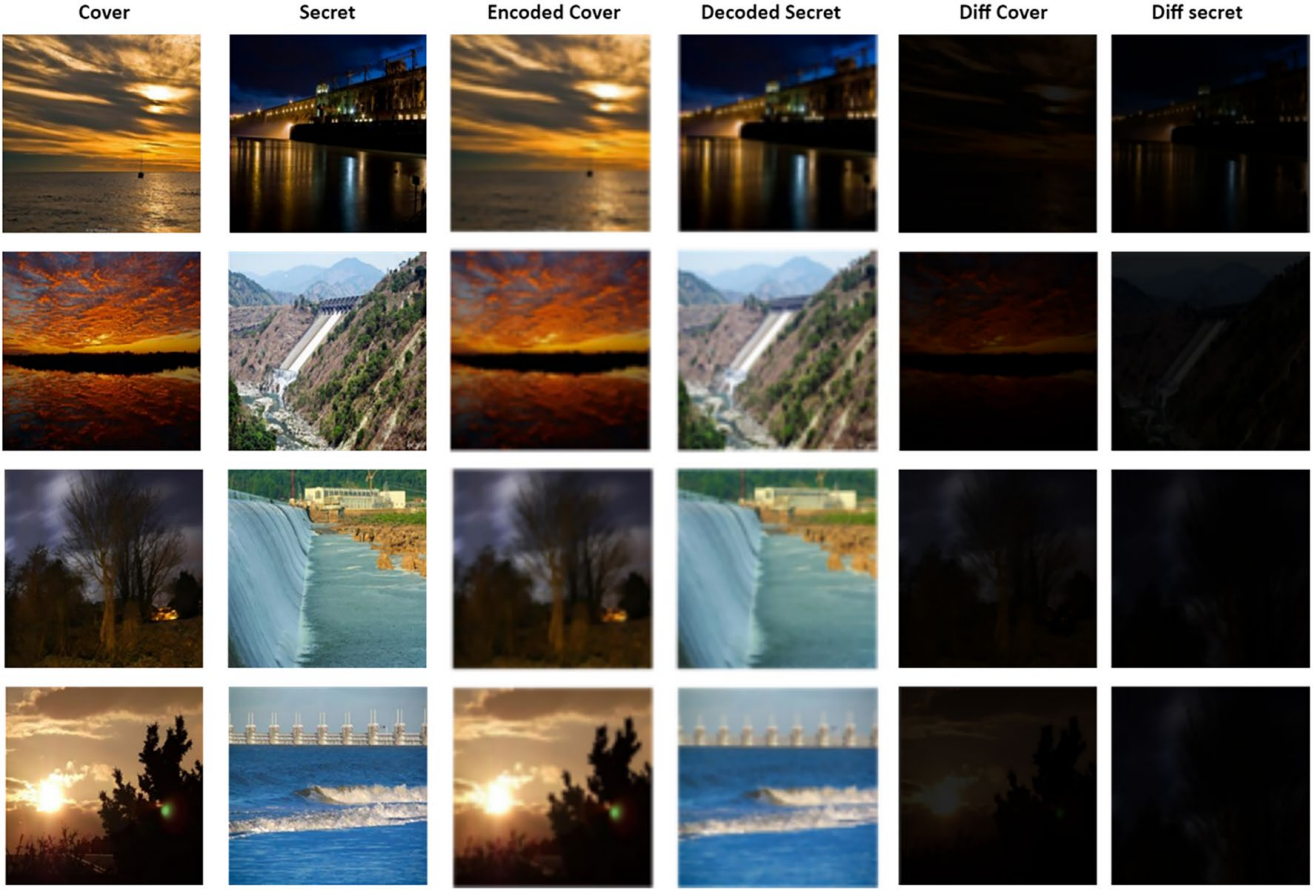
Test samples U-Net++ encoder model.

In this section, the sample results obtained by employing U-Net, V-Net, U-Net++ encoders and the decoder, designed in this research, are shown in Figs. 6, 7 and 8. For proving the efficacy of the encoders and decoder, the difference (diff cover) between the original and encoded cover image is calculated. Also, the difference (diff secret) between the original and decoded secret image is calculated. Both the differences are approximate to zero. Thus, plotting the pixels of the difference gave the black color image as shown in Figs. 6, 7 and 8.

**Figure 9:**
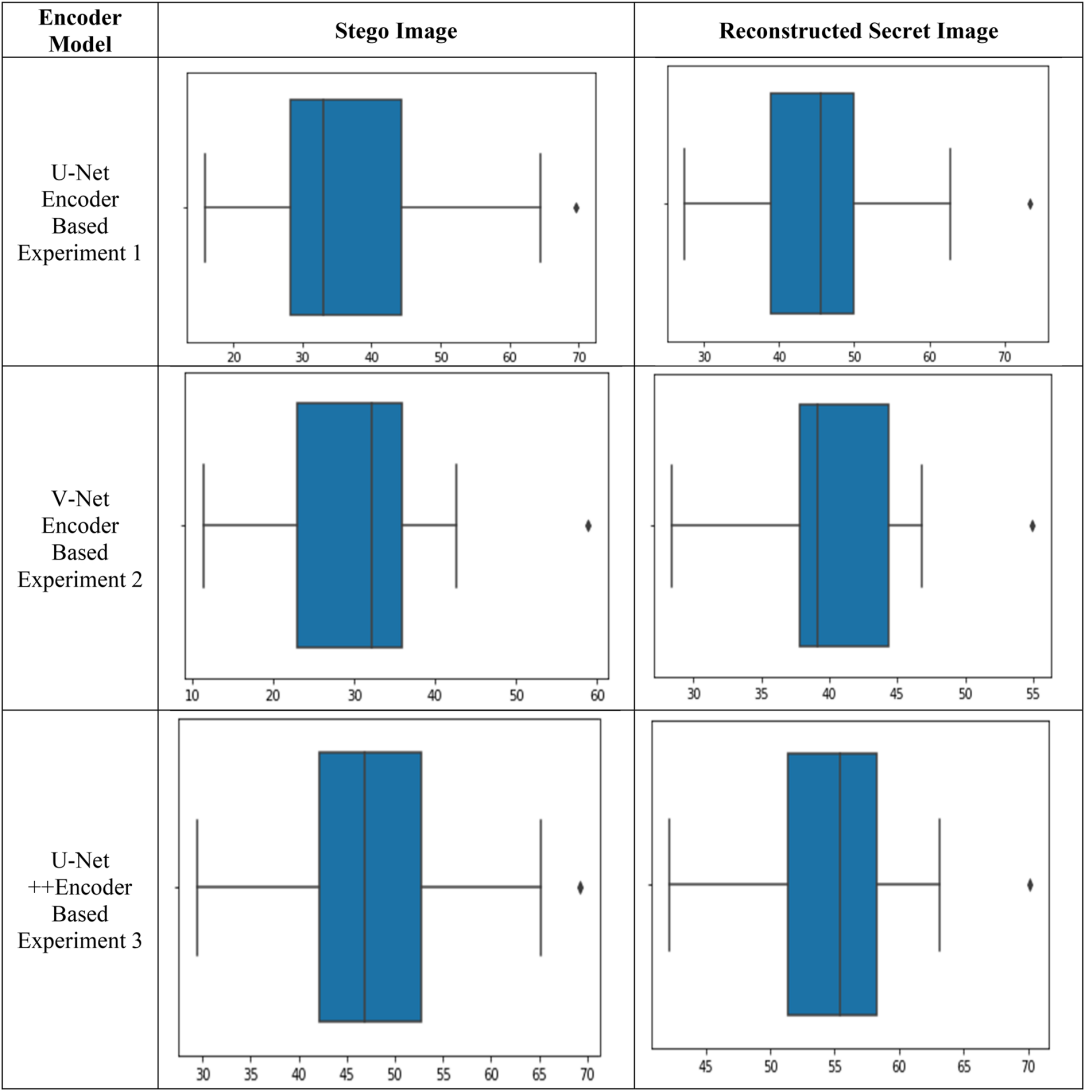
BRISQUE score scaling.

Further, the visual quality of the stego and reconstructed images is demonstrated in Tables 3, 4, 5, 6, and Fig. 9, respectively.

**Table 3:** Mean square error of Stego image and reconstructed secret image.

Encoder model	MSE	
	Cover and Stego image	Secret and reconstructed secret image
U-Net Encoder	0.0001	0.0003
V-Net Encoder	0.0019	0.0010
U-Net++ Encoder	0.007	0.006

**Table 4:** Peak signal to noise ratio of Stego and reconstructed secret image.

Encoder model	PSNR					
	Cover and Stego image			Secret and reconstructed secret image		
	Minimum	Maximum	Mean	Minimum	Maximum	Mean
U-Net encoder	35.00	41.02	38.00	29.00	38.94	38.00
V-Net encoder	27.80	31.20	30.00	30.20	34.40	33.00
U-Net++ encoder	18.50	29.00	24.00	21.00	33.40	27.00

**Table 5:** Structure similarity index of Stego and reconstructed secret image.

Encoder model	SSIM (%)					
	Cover and Stego image			Secret and reconstructed secret image		
	Minimum	Maximum	Mean	Minimum	Maximum	Mean
U-Net Encoder	90.00	99.40	98.75	89.74	99.89	98.69
V-Net Encoder	93.00	97.10	96.80	92.20	98.30	98.10
U-Net++ Encoder	88.00	95.40	91.00	85.00	95.50	93.00

**Table 6:** The entropy of Stego and reconstructed secret image.

Encoder model	Entropy								
	Original image			Stego image			Reconstructed secret image		
	Minimum	Maximum	Mean	Minimum	Maximum	Mean	Minimum	Maximum	Mean
U-Net Encoder	6.31	7.94	7.32	6.10	7.94	7.45	5.79	7.91	7.47
V-Net Encoder	6.31	7.94	7.32	7.00	7.91	7.59	5.3	7.85	7.40
U-Net++ Encoder	6.31	7.94	7.32	6.80	7.90	7.54	5.69	7.80	7.40

It is evident from the values of MSE shown in the first row of Table 3 that there is a difference of merely 0.0001 in the MSE of cover and stego images generated by the U-Net encoder and 0.0003 in the original and reconstructed secret images by the decoder. Further, it is clear from the results shown in the second row that the difference is 0.0019 for cover and stego images and 0.0010 for secret and reconstructed images when V-Net is employed as an Encoder. It is apparent from the third row that the difference for cover and stego image is 0.007 and 0.006, for secret and reconstructed images in the U-Net++ encoder model. Based on the comparison of the values of MSE presented in Table 3, it is observed that the U-Net encoder reports the minimum MSE. This proves the supremacy of U-Net based encoders over the V-Net and U-Net++ based encoders.

Further, the values of PSNR presented in Table 4 also showcase the error of reconstruction. In strong contrast to the MSE, higher PSNR indicates better quality of image reconstruction. For the stego image, the U-Net, V-Net, and U-Net++ report the highest PSNR of 41.02, 31.20, and 29.0 decibels, respectively. For the reconstructed secret image, U-Net, V-Net, and U-Net++ based architectures report the maximum values of 38.94, 34.40, and 33.40, respectively. It is apparent from these values that U-Net based architecture generates better quality images than V-Net and U-Net++ based architectures.

Now, the values of SSIM shown in Table 5, demonstrate the quality degradation caused during image reconstruction. The U-Net, V-Net, and U-Net++ based architectures report the highest similarity of 99.89%, 97.10%, and 95.40%, respectively, between stego and cover images. Similarly, for the reconstructed secret image, the values of SSIM are 99.40%, 98.30%, and 95.50% for the U-Net, V-Net, and U-Net++ based architectures, respectively.

It is clear from the values of SSIM that the U-Net based architecture generates the stego and reconstructed secret images with the highest degree of similarity. Thus, the generated images are approximately indistinguishable from their corresponding original images.

As shown in Table 6, the values of entropy are calculated to showcase the degree of randomness in the generated images. For the stego image, the U-Net, V-Net, and U-Net++ based architectures report the mean entropy of 7.94, 7.91, and 7.90, respectively. For the reconstructed secret images, these architectures give the mean entropy of 7.91, 7.85, and 7.80, respectively. It is evident from the given values that all the three networks generate images with a similar degree of randomness as the original images. These architectures generate images that retain the maximum information. Further, it is also observed that U-Net-based architecture gives the highest entropy values for both the stego and reconstructed secret images. Therefore, it outperforms the V-Net and U-Net++ based architectures in terms of retaining the information.

Now, the values of the BRISQUE score, as shown in Fig. 9, are calculated to prove the perceptual quality of the generated images. The lower values of the BRISQUE score favor the better perceptual quality of images. For the stego images, the U-Net, V-Net, and U-Net++ based architectures give the lowest BRISQUE score of 15.87, 11.40, and 29.44, respectively. For the reconstructed images the lowest values are 27.34, 28.39, and 42.18 for the U-Net, V-Net, and U-Net++ based architectures, respectively. These values indicate that the V-Net architecture outperforms the U-Net, and U-Net++ architectures in terms of the perceptual quality of generated images.

It is observed from the experimental results obtained that the U-Net based encoder generates high-quality stego and reconstructed secret images as compared to the other two encoder models. Further, the V-Net based encoder regenerated the images with good perceptual quality, Still, it lacks information preserving and maintaining the structural similarity between the generated images and their corresponding input images. It is also evident from the results shown in Tables 3, 4, 5, 6 and 7 and Fig. 9 that the U-Net++ encoder is a poor performer than U-Net and V-Net architectures in image steganography.

**Table 7:** BRISQUE Score of Stego and reconstructed secret image.

Encoder model	BRISQUE Score					
	Stego image			Reconstructed secret image		
	Minimum	Maximum	Mean	Minimum	Maximum	Mean
U-Net encoder	15.87	69.656	36.02	27.34	73.45	45.36
V-Net encoder	11.40	58.91	31.30	28.39	54.95	40.46
U-Net++ encoder	29.44	69.27	47.83	42.18	70.13	54.71

### Steganographic payload capacity

An efficient steganographic technique aims to embed maximum information into cover media without affecting the visual quality so that an attacker cannot percept it as a target image. The payload capacity is the embedding rate at which the number of secret data bits is embedded in the cover image, Table 8 depicts the comparison of payload capacity between proposed and existing techniques, here second and third column shows the size of the secret and cover image, respectively. The fourth column represents the relative payload capacity, calculated as per Eq. (13).

**Table 8:** Comparisons of steganographic payload capacity.

Encoder model	Secret image size (absolute capacity)	Cover image size	Relative payload capacity
U-Net encoder	256 × 256 × 3	256 × 256 × 3	1
V-Net encoder	256 × 256 × 3	256 × 256 × 3	1
U-Net++ encoder	256 × 256 × 3	256 × 256 × 3	1

13 $$\mathrm{Relative}\, \mathrm{Payload}\, \mathrm{Capacity}=\frac{\mathrm{Absolute}\, \mathrm{Capacity} }{\mathrm{Cover}\, \mathrm{Image}\, \mathrm{Size}}$$

Here, a secret color image of size 256 × 256 is embedded in the cover image of the same size. Hence, the relative payload capacity of all the three steganography techniques is 1 byte/pixel. In CNN-based steganographic methods^22,24^, a gray-scale secret image is embedded in the color cover image to maintain stego image quality. It can be observed from Tables 3, 4, 5, and 8, that the proposed techniques improve the steganographic payload capacity without compromising the image quality.

## Conclusions

In this paper, performance parameter assessment of deep learning-based image steganography techniques U-Net, V-Net, and U-Net++ based encoders are carried out. The encoder architectures generate the stego image that hides the secret image into the cover image. The unique and robust decoder is designed that effectively extracts the secret image from the stego image. The visual quality of the secret image reconstructed by the decoder is evaluated in terms of MSE, SSIM, PSNR, Entropy, and Brisque Score. It is observed from the comparative performance analysis of U-Net, V-Net, and U-Net++ based architectures that the U-Net architecture outperforms the other two architectures. This architecture ensures the high payload capacity without compromising the visual quality of reconstructed and stego images. Thus, it is useful for securing the data of real-life applications such as healthcare, defense, scientific documents, etc*.* Further, there is a vast scope of integrating the encryption and steganography techniques for enhancing security.

### Ethical statement

All methods were carried out in accordance with relevant guidelines and regulations.

## Data Availability

The Datasets analyzed during the current study are available for public access through Labeled Faces in the Wild^46^ and Know Your Data^47^.
